# Two Degree-of-Freedom Fiber-Coupled Heterodyne Grating Interferometer with Milli-Radian Operating Range of Rotation

**DOI:** 10.3390/s19143219

**Published:** 2019-07-22

**Authors:** Fuzhong Yang, Ming Zhang, Yu Zhu, Weinan Ye, Leijie Wang, Yizhou Xia

**Affiliations:** 1 State Key Laboratory of Tribology, Department of Mechanical Engineering, Tsinghua University, Beijing 100084, China; 2 Beijing Lab of Precision/Ultra-Precision Manufacture Equipment and Control, Tsinghua University, Beijing 100084, China

**Keywords:** grating interferometer, encoder, heterodyne, two-degree-of-freedom, fiber-coupled, signal contrast

## Abstract

In the displacement measurement of the wafer stage in lithography machines, signal quality is affected by the relative angular position between the encoder head and the grating. In this study, a two-degree-of-freedom fiber-coupled heterodyne grating interferometer with large operating range of rotation is presented. Fibers without fiber couplers are utilized to receive the interference beams for high-contrast signals under the circumstances of large angular displacement and ZEMAX ray tracing software simulation and experimental validation have been carried out. Meanwhile, a reference beam generated inside the encoder head is adopted to suppress the thermal drift of the interferometer. Experimental results prove that the proposed grating interferometer could realize sub-nanometer displacement measurement stability in both in-plane and out-of-plane directions, which is 0.246 nm and 0.465 nm of 3σ value respectively within 30 s.

## 1. Introduction

**Figure 1:**
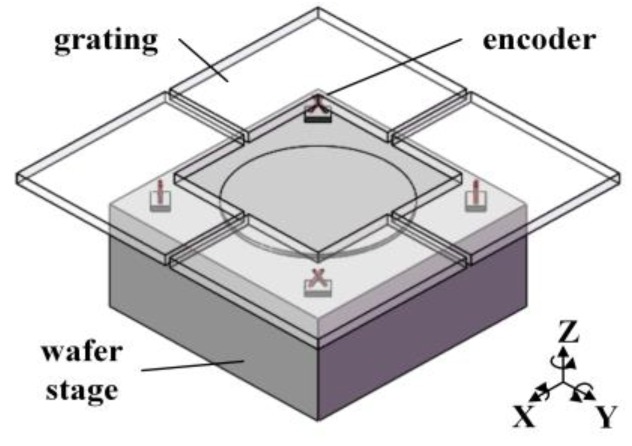
The schematic diagram of the encoder measurement system of the wafer stage.

In lithography machines for semiconductor fabrication, the laser interferometer and grating interferometer have been extensively applied due to their nanometer, even sub-nanometer, precision measurement ability [1,2,3]. Since the very beginning, the laser interferometers have served this industry and take the wavelength as measurement benchmark, which limits the measurement performance for their suffering from the noise of laser source frequency fluctuation, thermal drift and refractive index variation [4] even with a well controlled environment. By contrast, the grating interferometer, also known as interferometric encoder, is less subject to those impacts owing to the stability of grating period and short fixed optical length [5,6,7]. In the mainstream of 28 nm to 10 nm lithography equipment, the encoder head is fixed at each corner of the wafer stage, combined for the measurement of six-degree-of-freedom displacement relative to the grating (including a ±1 mm range in out-of-plane direction and ±1.5 mrad angular range in rotation) as Figure 1 shows. For ultra-precision measurement of the wafer stage, in-plane and out-of-plane displacement measurement ability in the motion range and sub-nanometer stability over the exposure time of a single wafer (given a throughput of more than 200 wafers per hour, this time is typically less than about half minutes) are demanded of a single interferometric encoder [2,3].

Compared with homodyne grating interferometers [8,9], heterodyne detection is highly insusceptible to direct current (DC) offset and amplitude variation of the output signals. Although frequency mixing caused by coaxial beams would lead to large periodic non-linearity error in heterodyne interferometric encoders, spatially separated beams can be utilized for this error reduction to tens of picometers typically [10,11,12]. Among previous works, the Physikalisch-Technische Bundesanstalt’s (PTB) design with single diffraction and a symmetric arrangement can achieve sub-nanometer stability with a 3σ value being 0.114 nm over 30 s in one-dimensional linear displacement measurement under ±50 mK temperature variation. Other symmetrical and quasi-common optical path designs are also adopted to minimize the environmental noise as much as possible [13,14]. However, in the lithography machines, encoders are combined for measuring the linear as well as the angular displacement of the wafer stage. Considering the inevitable misalignment between the encoder head and the grating as well as the angular displacements of the wafer stage, the contrast of the interference signals decreases severely, which would affect the measurement precision [15]. A corner reflector with double diffraction is one way of maintaining high signal contrast and increasing the operating range of rotation [16,17], but on the other hand, complex optical configuration restricts the compactness and increases the manufacturing difficulty of the encoder, exerting a critical influence on the installation and application in the wafer stage. Also, collimators used for returning light coupling would also increase whole volume of the encoder. Moreover, measurement noise induced by temperature variation on optical components should be taken into consideration, since thermal expansion or contraction would also result in large measurement errors [18].

In this paper, a two-degree-of-freedom fiber-coupled heterodyne grating interferometer with milli-radian operating range of rotation is proposed. The encoder with simple configuration employs fibers as the receiver of the interference beams to enhance the signal contrast. The reference beam inside the encoder head is designed to reduce the environmental noise. Therefore, measurement errors from thermal expansion are maximum minimized. Finally, experiment results demonstrate that this fiber-coupled heterodyne encoder can achieve sub-nanometer measurement stability with tens of pico-meter resolution.

## 2. Materials and Methods

### 2.1. Optical Configuration and Principle

**Figure 2:**
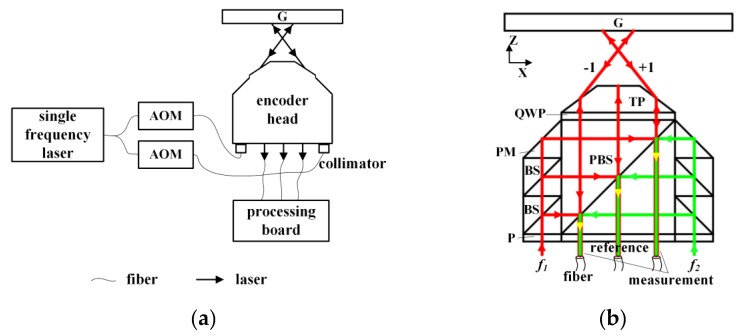
(**a**) Schematic of the two-degree-of-freedom fiber-coupled heterodyne grating interferometer (AOM: acousto-optical modulators, G: diffraction grating); (**b**) The optical configuration of the encoder (BS: beam splitter, P: polarizer, PBS: polarizing beam splitter, PM: prism mirror, QWP: quarter-wave plate, TP: trapezoidal prism).

The two-degree-of-freedom fiber-coupled heterodyne grating interferometer with a one-dimensional diffraction grating (G), proposed in this study, is shown in Figure 2a. The Littrow configuration is adopted since it is able to enlarge the out-of-plane displacement measurement range [13,16,19]. The beam generated by a single frequency laser with a wavelength of 780 nm is transported and split by single mode fibers. Two acousto-optical modulators (AOM) with modulation frequencies of 115 MHz and 95 MHz are used for a beat-frequency of 20 MHz. These two beams are delivered to the encoder head after collimators. Finally, interference beams obtained from the interferometer are converted to the measurement phase by the processing board as illustrated. As Figure 2b shows, both two collimated lasers with different frequencies, *f*_1_, *f*_2_, are S-polarized by polarizers (P) and divided into three beams by the beam splitters (BS). Beams with frequency *f*_1_ are reflected by the polarizing beam splitter (PBS), and go through the quarter-wave plate (QWP). The beams marked as ±1 (±1 represents the diffraction order relative to the coordinate system) are refracted by the trapezoidal prism (TP) and then diffracted back by the reflective grating, while the beam in the middle is reflected back by reflective coating on the top of the TP. They are combined with the beams with frequency *f*_2_ by PBS and interfered at P. All the interference beams are obtained and transported by multimode fibers without fiber couplers, of which the core diameter is much smaller than the size of the beam spot. Interference beams on both sides are used for the displacement measurement in *x* and *z* directions of the grating and the middle beam is taken as the reference for phase extraction.

Taking no account of any error, the relation between the phase changes and the two-degree-of-freedom displacement, Δ*x* and Δ*z*, can be expressed as:

(1) $$\Delta \phi_{+1}=-2\pi \left(\frac{+1}{p}\Delta x+\frac{2\cos\theta }{\lambda }\Delta z\right),$$

(2) $$\Delta \phi_{-1}=-2\pi \left(\frac{-1}{p}\Delta x+\frac{2\cos\theta }{\lambda }\Delta z\right),$$

where, Δ*ϕ* represents the detected phase changes, *p* is the grating period, *λ* is the wavelength, and *θ* is the Littrow angle, which can be obtained from the grating equation and expressed by:

(3) $$\theta =\arcsin\frac{\lambda }{2p},$$

Thus, the in-plane and out-of-plane displacement can be decoupled by:

(4) $$\Delta x=\frac{p(\Delta \phi_{-1}-\Delta \phi_{+1})}{4\pi },$$

(5) $$\Delta z=\frac{\lambda (\Delta \phi_{-1}+\Delta \phi_{+1})}{8\pi \cos\theta },$$

**Figure 3:**
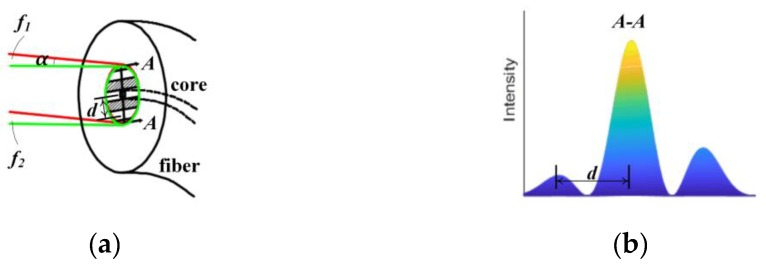
(**a**) The fiber without fiber coupler adopted for receiving the interference beams; (**b**) Intensity distribution diagram in the A–A cross-section.

To increase the operating range of rotation and maximize the signal contrast of the encoder, fibers without fiber couplers are used for enhancing contrast of the interference signals. As Figure 3a shows, when relative angular deflection between the grating and the encoder head exists, beams with frequency *f*_1_ and *f*_2_ would not coincide with each other and interference fringes will show up on the detected area. Figure 3b shows the intensity distribution diagram in one cross-section, A–A, perpendicular to the interference fringe. The parts with high and low intensity correspond to the bright and dark fringes in Figure 3a, respectively. After integration in the whole area, much of the alternating current (AC) component will change into the DC component. Under this circumstance, low signal contrast will be obtained by fiber couplers adopted for receiving interference beams.

Considering the wavelength *λ* of the laser and the angle *α* between the beams with frequencies *f*_1_ and *f*_2_, the fringe period *d* can be expressed as:

(6) $$d=\frac{\lambda }{\sin\alpha },$$

However, with no couplers used, fibers with a small core diameter, for example half of the fringe period, will receive only a little part of interference beams as shown in Figure 3a, which will reduce much DC component as well as the demand of the photodetectors. Considering the Gaussian beam profile, signal contrast cannot be maintained at 100%, but high values can still be acquired despite the interference fringes induced by large angular deflection. With enough output power of the laser source, signals with high quality can be acquired. The core diameter of the multimode fibers in our design is 0.2 mm. High-contrast measurement signals and large operating range of rotation can be obtained without retroreflector and double diffraction configuration.

**Figure 4:**
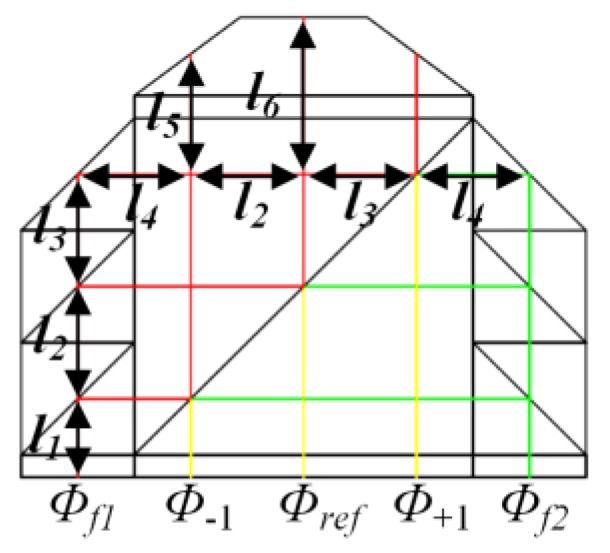
The sketch of the light path inside the encoder head.

Despite the asymmetrical optical length between the beams with different frequencies, a reference beam is generated inside the encoder head to minimize the measurement error induced by the expansion or contraction of the optical components. As Figure 4 shows, given uniform temperature variation in the encoder head and no displacement of the grating, the phase changes of all the interference signals caused by thermal drift, *ϕ*_±1, *ref*_, can be expressed by:

(7) $$\phi_{+1}=\left(\phi_{f1}+2\pi \frac{2\Delta l_{1}+3\Delta l_{2}+3\Delta l_{3}+\Delta l_{4}+2\Delta l_{5}}{\lambda }\right)-\left(\phi_{f2}+2\pi \frac{2\Delta l_{1}+2\Delta l_{2}+2\Delta l_{3}+\Delta l_{4}}{\lambda }\right),$$

(8) $$\phi_{-1}=\left(\phi_{f1}+2\pi \frac{2\Delta l_{1}+2\Delta l_{2}+2\Delta l_{3}+\Delta l_{4}+2\Delta l_{5}}{\lambda }\right)-\left(\phi_{f2}+2\pi \frac{2\Delta l_{1}+\Delta l_{2}+\Delta l_{3}+\Delta l_{4}}{\lambda }\right),$$

(9) $$\phi_{ref}=\left(\phi_{f1}+2\pi \frac{2\Delta l_{1}+3\Delta l_{2}+2\Delta l_{3}+\Delta l_{4}+2\Delta l_{6}}{\lambda }\right)-\left(\phi_{f2}+2\pi \frac{2\Delta l_{1}+2\Delta l_{2}+\Delta l_{3}+\Delta l_{4}}{\lambda }\right),$$

where, Δ*l*_1–6_ is the optical length changes in each section of the encoder and *ϕ _f_*_1, *f*2_ represents the initial phase of laser with frequencies *f*_1_ and *f*_2_. By subtracting the phase changes of the reference beam, the detected phase changes can be obtained as:

(10) $$\Delta \phi_{+1}=\phi_{+1}-\phi_{ref}=4\pi \frac{\Delta l_{5}-\Delta l_{6}}{\lambda },$$

(11) $$\Delta \phi_{-1}=\phi_{-1}-\phi_{ref}=4\pi \frac{\Delta l_{5}-\Delta l_{6}}{\lambda },$$

Combined with Equations (4) and (5), we can see that the measurement error caused by thermal drift of the encoder head in the *x* direction equals to zero and the error in *z* direction can be expressed as:

(12) $$\Delta z=\frac{\Delta l_{5}-\Delta l_{6}}{\cos\theta },$$

With a short optical length difference, thermal drift error in the out-of-plane direction can be easily controlled to sub-nanometer scale. In our design, this optical length difference is about 3 mm. In view of 0.01 °C temperature variation in the application of lithography machines, the error caused by expansion or contraction of the optical encoder is about 0.213 nm with the coefficient of thermal expansion (CTE) of BK7 glass being 7.1 × 10^−6^, and substituted by fused silica with the CTE being 0.55 × 10^−6^, the error will be less than 0.017 nm.

### 2.2. Simulation

**Figure 5:**
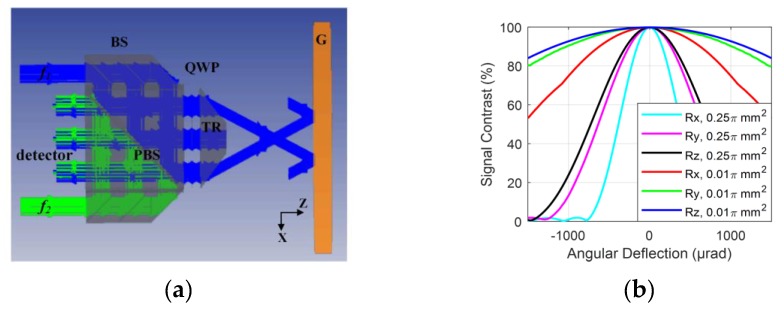
(**a**) Simulation model set in ZEMAX; (**b**) simulation result of the signal contrast with angular deflection varying from −1.5 mrad to 1.5 mrad in three directions of rotation.

To detail the large operating range of rotation and high signal contrast in our structure proposed, the simulations are made based on the ZEMAX ray tracing software operating in a non-sequential mode. The simulation model is shown in Figure 5a, which corresponds to the optical configuration in Figure 2b. Two incident beams are represented by frequency *f_1_* and *f_2_* respectively. The wavelength set in the model is 780 nm and the grating has grooves density of 1200 lines/mm, which equals to a grating period of 833.33 nm. The beam diameter is 1mm and the detectors with areas of 0.25π mm^2^ and 0.01π mm^2^ are respectively employed to acquire the intensity of interference beams. The power of incident beams with frequencies *f*_1_ and *f*_2_ is set equal on detectors and the angular deflection of the grating is set by the interactive extension with MATLAB in all three directions.

As shown in Figure 5b, compared with the simulation result with areas of 0.25π mm^2^, the signal contrast on a detected area of 0.01π mm^2^ decreases slowly as the angular deflection enlarges. The most severe reduction of the contrast is about 46.9% with 1.5 mrad angular deflection around *x*-axis, which is demanded in the application of lithorgraphy machines. The remaining values are still high enough for phase extraction. Thus, milli-radian operating range of rotation with enough signal contrast can be obtained by removing the fiber coupler for the receiving of the interference beams.

## 3. Results and Discussion

### 3.1. Experimental Setup

The followed experiments were carried out to demonstrate the performance of the grating interferometer presented. In the experiments, a reflective diffraction grating (GH25-12V, Thorlabs, NJ, USA) is adopted and the grating period is 833.33 nm. The wavelength of the single frequency laser is 780 nm. Two AOMs (MT110, AA Opto Electronic, Orsay, France) are used to modulate the frequencies. The optical components in the encoder head are bonded together and the core diameter of the multimode fibers employed is 200 μm. Since the fibers are used without the couplers, the intensity of the light received as well as the measurement phase is relatively susceptible to the changes of fibers’ position and orientation during coupling. It is necessary to fix and adjust the fibers well. Moreover, the output power of the laser with proper beam diameter should be increased due to the low power of the signals caused by the reduced detection area. All the interference beams are transmitted to photodetectors on the processing board (ZMI 4100TM Series Measurement Board, ZYGO, Connecticut, USA) by fibers without fiber couplers, and the measurement phases obtained are off-line processed by MATLAB on a personal computer. Considering the electronic subdivision of measurement board being 8192, the theoretical resolution in both measurement directions, Δ*x_res_* and Δ*z_res_*, achieves tens of pico-meter resolution, which can be calculated according to,

(13) $$\Delta x_{res}=\frac{p\Delta \phi_{res}}{4\pi }=0.051nm,$$

(14) $$\Delta z_{res}=\frac{\lambda \Delta \phi_{res}}{8\pi \cos\theta }=0.027nm,$$

where, Δ*ϕ_res_* represents the phase resolution, which equals to 2π/8192 rad.

### 3.2. Large Operating Range of Rotation

**Figure 6:**
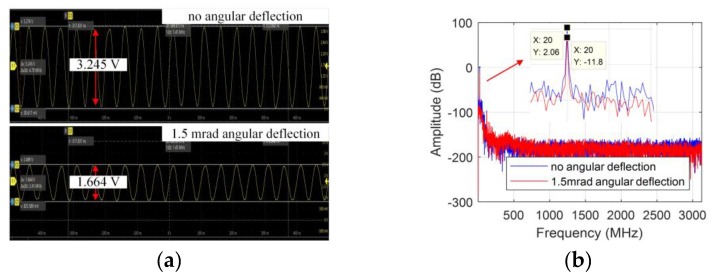
The interference signals obtained by the multimode fibers without fiber couplers: (**a**) waveforms; (**b**) the amplitude spectrum.

To verify a large operating range of rotation of the encoder, the interference beams obtained by the multimode fibers without fiber couplers are sent into the photodetectors (APD130A2, Thorlabs, NJ, USA), with a digital oscilloscope monitoring the waveforms of the interference signals. Initially, the alignment of the encoder as well as the power of beams interfered at the detectors are adjusted for high signal contrast as much as possible and then the relative deflection angle is realized by a 6-axis precision piezo stage (P-587, Physik Instrumente, Karlsruhe, Germany). One of the measurement signal waveforms is shown as Figure 6a and the amplitude spectrum is drawn in Figure 6b.

From the waveforms of the interference signal in Figure 6a, it can be seen that the AC component reduces about 1.581 V. The signal loss is about 13.14 dB according to Figure 6b. The contrast decreases from about 98.15% to 50.18% for the measurement interference signal when there is 1.5 mrad angular deflection between the encoder head and the grating around the x-axis, which is consistent with the simulation. Thus, large operating range of rotation and signal contrast of the interferometer with single diffraction is demonstrated.

### 3.3. Measurement Stability

**Figure 7:**
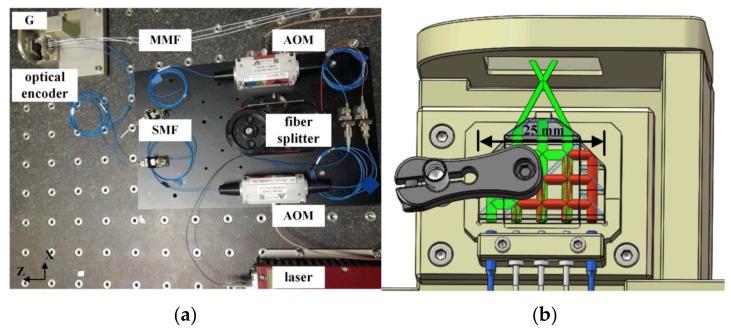
(**a**) The overall perspective of experiment setup (SMF: single mode fiber; MMF, multimode fiber); (**b**) the assembly model of the encoder head.

To validate the measurement stability of the interferometer, further experiment has been carried out. The overall perspective of the experiment setup is shown in Figure 7a. The grating is fixed together with the encoder head on a custom-made mounting as Figure 7b shows, which is in order to eliminate the influence of the mechanical vibration as much as possible. The finite element method (FEM) analysis shows the first nature frequency of the structure is above 4 kHz and all the devices are placed on a vibration isolation optical table for reduction of external vibration. Theoretically, no displacement below 4 kHz should be observed.

**Figure 8:**
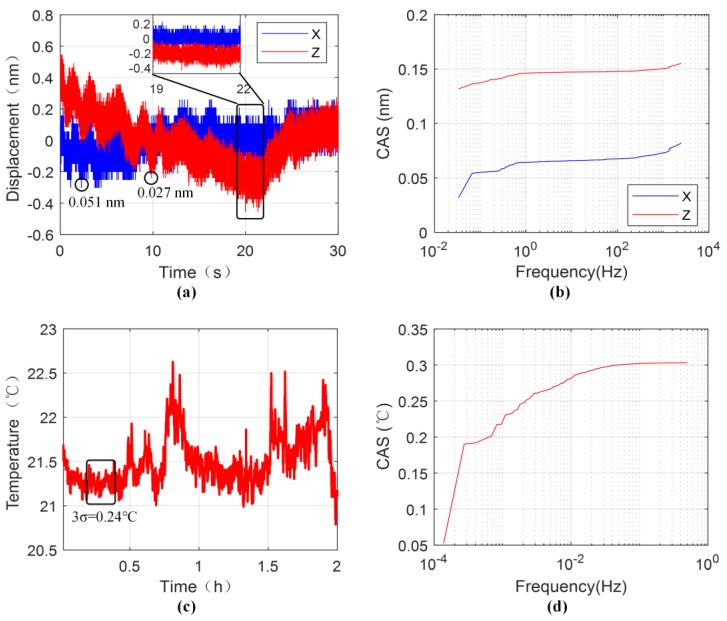
The measurement results of the grating interferometer: (**a**) Measurement stability in 30 s; (**b**) the cumulative amplitude spectrum (CAS) of Figure 8a; (**c**) Environmental temperature fluctuation in 2 h; (**d**) the CAS of Figure 8c.

Figure 8a shows the displacement measurement results of the grating interferometer with a sampling rate of 5 kHz. Considering the application of lithography machines, the results are collected over 30 seconds. Along with the resolution of 0.051 nm and 0.027 nm, the 3*σ* values of the results are 0.246 nm and 0.465 nm in *x* and *z* directions, respectively. As can be seen in Figure 8b, the noise below 0.1 Hz frequency takes up the majority of the measurement error in the cumulative amplitude spectrum. Figure 8c shows the temperature fluctuation of the experiment environment in 2 h with a sampling rate of 1 Hz, and the 3σ value in 10 min is about 0.24 °C. Similarly, low frequency noise accounts for the most of the temperature fluctuation, shown in Figure 8d. Therefore, the reason for the drifts of the measurement result in the *z* direction may largely be attributed to the environment influence. After a 0.1 Hz high-pass filter, the 3σ values of the measurement results are 0.080 nm and 0.053 nm in the x and z directions, which can be ascribed to the electronic noise of the processing board. Meanwhile, considering of better stability of the grating period and no thermal drift of the encoder in theory, the impact in the x direction is relatively smaller. As shown in the close-up over 3 s of Figure 8a, the measurement results are little affected and the 3σ values in the *x* and *z* directions, 0.150 nm and 0.147 nm, and further performance enhancement of the encoder can be realized by stricter environmental control in the lithography machines.

## 4. Conclusions

A two-degree-of-freedom fiber-coupled heterodyne grating interferometer with milli-radian operating range of rotation is presented and tested. To acquire high-contrast measurement signals in milli-radian operating range of rotation, fibers without fiber couplers for receiving the interference beams are proposed. The thermal drift of the encoder head is analyzed and a reference beam is employed to suppress the error and enhance the measurement stability. The experimental results show that, within 30 s, the 3σ values of displacement measurement in the *x* and *z* directions achieve 0.246 nm and 0.465 nm respectively, which realizes the requirement of sub-nanometer stability in the application of lithography machines.
